# Vortex-like vs. turbulent mixing of a* Viscum album* preparation affects crystalline structures formed in dried droplets

**DOI:** 10.1038/s41598-024-63797-z

**Published:** 2024-06-05

**Authors:** Maria Olga Kokornaczyk, Carlos Acuña, Alfonso Mier y Terán, Mario Castelán, Stephan Baumgartner

**Affiliations:** 1 Society for Cancer Research, 4144 Arlesheim, Switzerland; 2 Institute for Complementary and Integrative Medicine, University of Bern, Freiburgstrasse 40, 3010 Bern, Switzerland; 3 Robotics and Advanced Manufacturing, Center for Research and Advanced Studies of the National Polytechnic Institute, 25900 Ramos Arizpe, Mexico; 4 Institute of Integrative Medicine, University of Witten-Herdecke, 58313 Herdecke, Germany

**Keywords:** Crystallization, Turbulent and laminar flow, Droplet evaporation, Homeopathy, Deep-learning, Medical research, Chemistry, Materials science

## Abstract

Various types of motion introduced into a solution can affect, among other factors, the alignment and positioning of molecules, the agglomeration of large molecules, oxidation processes, and the production of microparticles and microbubbles. We employed turbulent mixing vs. laminar flow induced by a vortex vs. diffusion-based mixing during the production of *Viscum album Quercus* L. 10^−3^ following the guidelines for manufacturing homeopathic preparations. The differently mixed preparation variants were analyzed using the droplet evaporation method. The crystalline structures formed in dried droplets were photographed and analyzed using computer-supported image analysis and deep learning. Computer-supported evaluation and deep learning revealed that the patterns of the variant succussed under turbulence are characterized by lower complexity, whereas those obtained from the vortex-mixed variant are characterized by greater complexity compared to the diffusion-based mixed control variant. The droplet evaporation method could provide a relatively inexpensive means of testing the effects of liquid flow and serve as an alternative to currently used methods.

## Introduction

Investigating the impact of succussion on a pharmaceutical preparation, detecting the succussion’s modality, or proving its occurrence represent challenging tasks for research and quality control of medicinal products.

Interestingly, different branches of medicine hold varying views on the importance of succussing medicinal solutions. In allopathic medicine, even an accidental agitation of a preparation is avoided, as it is considered a risk factor for reducing the therapeutic properties of the solution by altering the protein structure or increasing oxidation processes^1–3^. In contrast, in homeopathy, as also in anthroposophical pharmaceutics, which employ the potentization procedure (i.e., subsequent dilutions and succussions performed until reaching the desired potency), agitation represents an integral part of the production protocol that is believed to determine the therapeutical properties of the preparation^4–6^, rather of reducing them.

The succussion performed during the potentization process may be characterized by its (1) modality, which refers to the type of movement used to induce motion into the solution; (2) duration, indicating the time or quantity of movements performed during one potentization step; and (3) performance, distinguishing between handmade or machine-made agitation, or sonication^6^.

Among the different producers of homeopathic and anthroposophic preparations, the applied succussion techniques vary greatly regarding the abovementioned characteristics. Generally, producers following the Hahnemannian guidelines perform turbulent agitation by hitting the flask against an elastic surface, which can be done by hand or machine^7,8^. In contrast, producers of anthroposophical products induce an ordered motion, such as vortex- or lemniscate-like flow, or an alteration of ordered and turbulent motion (i.e., ordered vortex-like flow interrupted by turbulence and then continuing, or a sequence of vortexes turned into left and right with turbulence while changing direction)^9^. Additionally, it can be generalized that low potencies, primarily used in anthroposophic medicine, are potentized by ordered motions, while high and ultra-high potencies, mainly applied in classical homeopathy, are produced using turbulent flow.

A series of previous experiments demonstrated that self-assembled patterns formed in droplets during drying serve as a suitable tool for investigating low dilutions. Characteristics of the self-assembled structures, such as grey level distribution, texture, and fractal features, were used as output parameters. The parameters proved to be substance-specific, allowing differentiation between dilutions prepared from different substances up to their fourth decimal dilution^10^. They were also sensitive to the effects of vertical vigorous shaking^5^ and interactions occurring among the components of complex preparations^11^.

In the present study, we applied the droplet evaporation method to obtain patterns from *Viscum album Quercus* L. 10^−3^ (*VaQ* 3×) produced following the guidelines for manufacturing homeopathic preparations^7^ by applying three different mixing modalities following each of the three decimal dilution steps: (1) turbulent vertical machine-made succussions (variant T), (2) laminar flow induced by a handmade vortex (variant L; Supplement 1) and (3) diffusion-based mixing (unsuccussed control, variant D).

The obtained patterns underwent evaluation using computer software and deep learning algorithms. Advanced semi-supervised and unsupervised deep learning models were employed, known for their proficiency in understanding and identifying intricate patterns that may elude classical analysis methods. The semi-supervised algorithm showcased its versatility by accommodating the mixing modalities into three predetermined clusters. On the other hand, the unsupervised learning model exhibited its capability to distinguish a broader array of pattern families with shared texture similarities. It leveraged advanced texture feature extraction from Deep Texture Representation matrices and automatically determined 13 clusters. This allowed for a more precise characterization and differentiation of the mixing modalities compared to the semi-supervised approach.

## Results

### Visual pattern assessment

**Figure 1:**
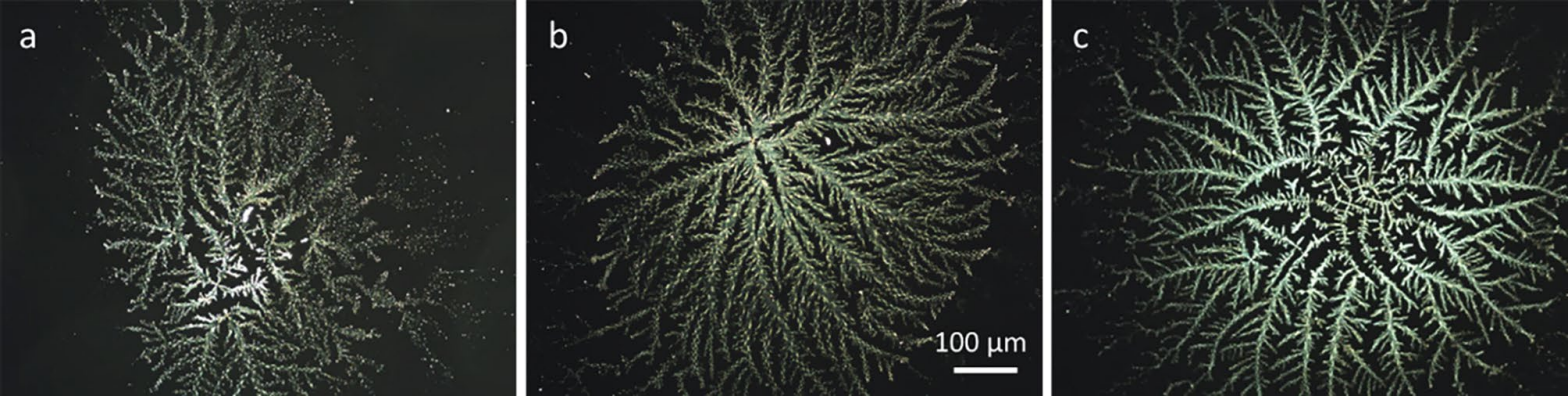
Examples of central structures formed inside dried droplets of Viscum album Quercus L. 3× variants prepared by different mixing techniques: machine-made turbulent succussions (**a**), laminar flow induced by handmade vortex (**b**), and diffusion-based mixing (**c**). Images with local connected fractal dimension equal to or similar to that of the variant’s mean are presented. Photographs were taken in darkfield and magnification of 100×.

The droplet residues from all tested variants of differently mixed *Viscum album Quercus* L. 3× (*VaQ* 3×) variants exhibited dendritic, fractal structures in their central regions, most likely formed during the diffusion-limited aggregation of particles occurring during droplet evaporation (Fig. 1). These central structures appeared bright, with no other shapes visible within the droplet remnants. In most patterns, there was a distinct crystallization center or region from which the longest and thickest first-order branches extended toward the outer region of the structure. In addition, there were shorter and thinner higher-order branches. These branches appeared bushy and were covered with needles. In most cases, variant T (mixed by induction of turbulent flow; see Fig. 1a) formed smaller and less complex central structures compared to variants L and D (mixed by laminar flow and diffusion-based mixing, respectively). The difference between the latter two variants was difficult to detect visually (see Fig. 1b,c).

### Computerized pattern evaluation

The results of the computerized pattern evaluation are shown in Table 1. All analyzed pattern evaluation parameters describing the grey level distribution, texture, and fractality of the structures were able to differentiate significantly between variant T and the other two variants, whereas four parameters, *mass fractal dimension*, *fractal dimension of structures with highest value r2*, *ascending second moment* and *inverse difference moment* were able to differentiate significantly between all three variants. In these cases, the most significant difference was observed between variants T and L, while variant D exhibited minor differences compared to either L or T.

**Table 1:** On the left side: Mean values of analyzed pattern evaluation parameters for the three differently mixed *Viscum album Quercus L.* 3× variants (T: turbulent, L: laminar, and D: diffusion-based) analyzed in the main experiments and three control groups analyzed in the systematic control experiments. On the right side: results of the 2-way analysis of variance with independent factors mixing procedure (Mixing) and experimentation day (Day). Mean values with different letter codes (a, b, c) are significantly different (p < 0.05).

Parameter	Mixing	Main experiments		SCE			Main experiments		SCE	
		n	Mean	n	Mean		F	*p*	F	*p*
LCFD	T	202	0.85 (b)	181	0.74 (a)	Mixing	55.9	<0.0001***	1.37	0.2557 ns
	L	193	1.21 (a)	179	0.82 (a)	Day	24.06	<0.0001***	19.26	<0.0001***
	D	204	1.17 (a)	182	0.81 (a)	Mixing*Day	3.8	0.0002***	2.99	0.0027**
MFD	T	202	− 1.54 (a)	181	− 1.45 (ab)	Mixing	33.59	<0.0001***	4.72	0.0093**
	L	193	− 1.69 (c)	179	− 1.47 (b)	Day	20.87	<0.0001***	32.38	<0.0001***
	D	204	− 1.65 (b)	182	− 1.4 (a)	Mixing*Day	5.82	<0.0001***	4.45	<0.0001***
D with highest r2	T	202	1.24 (c)	181	1.08 (a)	Mixing	42.44	<0.0001***	2.46	0.0862 ns
	L	193	1.58 (a)	179	1.18 (a)	Day	14.08	<0.0001***	27.7	<0.0001***
	D	204	1.5 (b)	182	1.13 (a)	Mixing*Day	3.51	0.0006***	3.01	0.08626**
LAC	T	202	0.37 (a)	181	0.36 (a)	Mixing	26.39	<0.0001***	0.35	0.7035 ns
	L	193	0.28 (b)	179	0.36 (a)	Day	17.42	<0.0001***	5.66	0.0002***
	D	204	0.27 (b)	182	0.34 (a)	Mixing*Day	6.11	<0.0001***	1.32	0.2305 ns
GLD	T	206	11.84 (b)	198	11.74 (a)	Mixing	37.95	<0.0001***	2.95	0.0532 ns
	L	196	16.18 (a)	197	13.08 (a)	Day	41.96	<0.0001***	25.06	<0.0001***
	D	204	16.46 (a)	198	11.64 (a)	Mixing*Day	3.24	0.0013**	3.43	0.0007***
ASM	T	206	0.021 (a)	198	0.022 (b)	Mixing	16.98	<0.0001***	5.02	0.0069**
	L	196	0.016 (c)	197	0.021 (b)	Day	84.67	<0.0001***	56.66	<0.0001***
	D	204	0.018 (b)	198	0.024 (a)	Mixing*Day	6.59	<0.0001***	2.07	0.0368*
Contrast	T	206	518.27 (b)	198	547.68 (a)	Mixing	32.47	<0.0001***	2.26	0.1053 ns
	L	196	752.57 (a)	197	637.62 (a)	Day	36.8	<0.0001***	21.05	<0.0001***
	D	204	810.62 (a)	198	557.88 (a)	Mixing*Day	5.71	<0.0001***	4.71	<0.0001***
Correlation	T	206	0.0015 (a)	198	0.0023 (a)	Mixing	19.27	<0.0001***	0.24	0.7824 ns
	L	196	0.0010 (b)	197	0.0021 (a)	Day	11.08	<0.0001***	37.55	<0.0001***
	D	204	0.0008 (b)	198	0.0023 (a)	Mixing*Day	4.59	<0.0001***	1.23	0.2786 ns
IDM	T	206	0.38 (a)	198	0.40 (b)	Mixing	22.36	<0.0001***	4.78	0.0087**
	L	196	0.35 (c)	197	0.39 (b)	Day	119.01	<0.0001***	42.98	<0.0001***
	D	204	0.36 (b)	198	0.41 (a)	Mixing*Day	4.99	<0.0001***	1.81	0.0728 ns
Entropy	T	206	5.64 (b)	198	5.49 (ab)	Mixing	31.23	<0.0001***	3.69	0.0256*
	L	196	6.17 (a)	197	5.57 (a)	Day	42.14	<0.0001***	43.95	<0.0001***
	D	204	6.06 (a)	198	5.34 (b)	Mixing*Day	4.05	<0.0001***	2.48	0.0118*

LCFD—local connected fractal dimension; MFD—mass fractal dimension; LAC—lacunarity; ASM—ascending second moment; IDM—inverse difference moment; SCE—systematic control experiments; *p < 0.05; **p < 0.001; ***p < 0.0001; ns—not significant.

The systematic control experiments detected no significances between the control groups for six out of ten (6/10) analyzed parameters or detected significances with p-values much smaller than those of the corresponding main experiments for 4/10 parameters. Thus, it can be assumed that the experimental system was reasonably stable, and the differences observed in the main experiments were primarily due to the applied mixing procedures. In the main experiments, the factor day (i.e., the influence of the experimentation day) was strongly significant for all ten parameters; the interaction between day and mixing procedure was also significant, however, with a much smaller p-value than that of the factor mixing procedure.

### Deep learning based pattern evaluation

The pattern evaluation results based on deep learning deriving from the supervised and unsupervised approach are described in detail elsewhere^12,13^.

**Figure 2:**
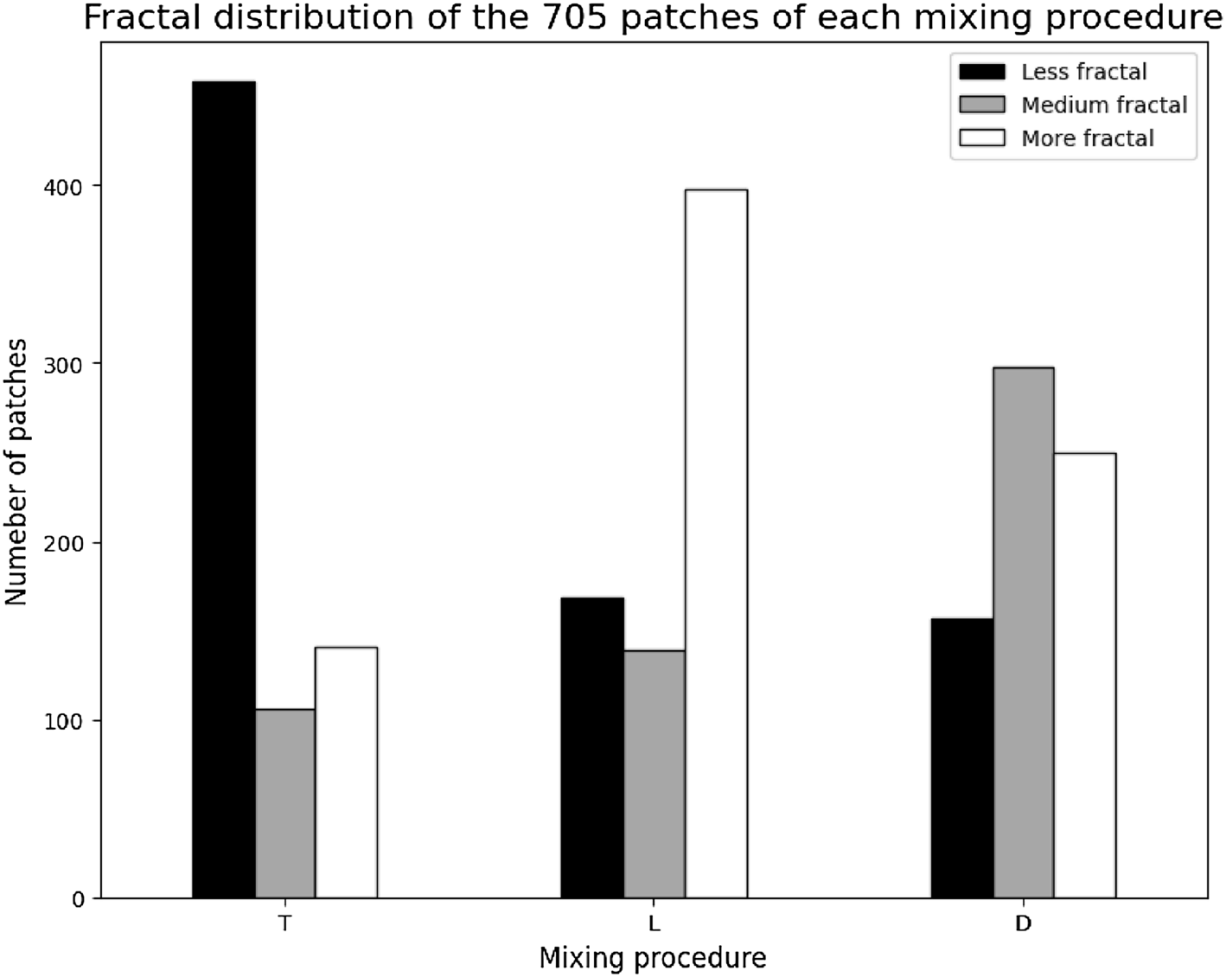
Distribution of image patches grouped after applying a semi-supervised deep learning approach for categories less fractal, medium fractal, and more fractal found in the patterns from dried droplets of *Viscum album Quercus* 3× produced with different mixing procedures: diffusion-based mixing (D), turbulent vertical succussions (T), and laminar flow induced by handmade vortex (L). The patches obtained from the L mixing procedure show the highest fractal composition, having 56.60% of the patches in the “more fractal” category. The patches obtained from the T mixing procedure exhibit the lowest fractal composition, having 65.2% of the patches in the “less fractal” category.

**Figure 3:**
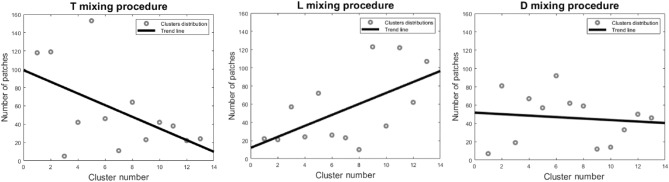
Distribution of image patches grouped after applying an unsupervised deep learning approach for categories less fractal (closer to 0 along the x-axis), medium fractal (closer to 7 along the x-axis), and more fractal (closer to 13 along the x-axis) found in the patterns from dried droplets of *Viscum album Quercus* 3× produced with different mixing procedures: D—diffusion-based mixing (middle), T—turbulent vertical succussions (left), L—laminar flow induced by handmade vortex (right). The 13 groups obtained from the unsupervised approach emphasize the fractal tendency already exhibited in Fig. 2 for the different mixing methods.

**Figure 4:**
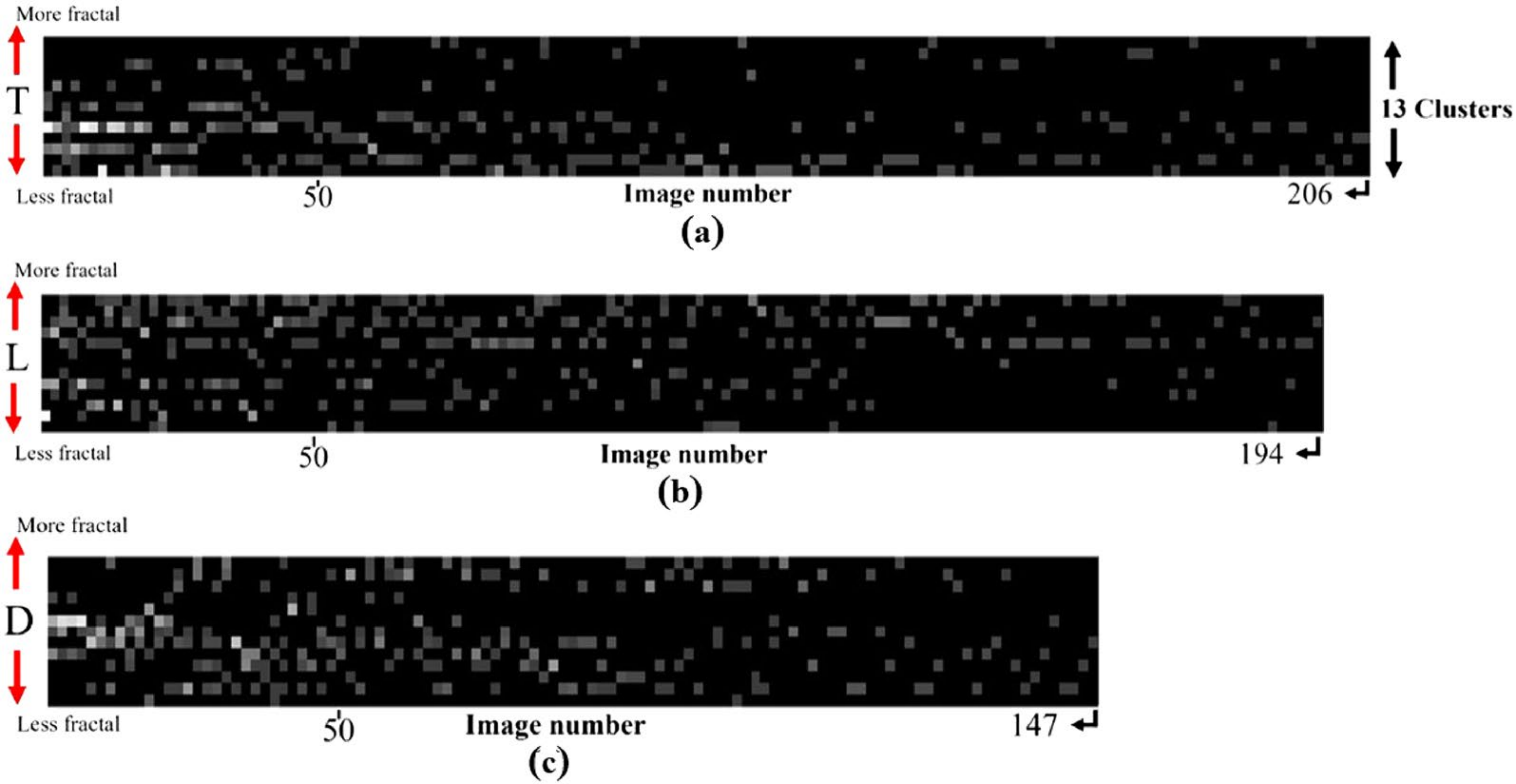
Patch distribution at image level for images obtained from unsupervised deep learning applied on dried droplets of *Viscum album Quercus* 3× produced by means of diffusion-based mixing (variant D) (**a**), turbulent mixing (variant T) (**b**), and laminar flow induced by a handmade vortex (variant L) (**c**). The bias toward more and less fractal behavior of the dried droplet is visible for the laminar and turbulent modalities, while the diffusion modality presents both biases.

Summarizing, both approaches revealed that the patterns obtained from dried droplets of the turbulently mixed variant T had a less fractal composition in comparison to the diffusion-based mixed control variant D. In contrast, the patterns obtained from variant L, mixed using the laminar flow, had a more fractal composition when compared to the control variant (Figs. 2, 3, 4).

**Figure 5:**
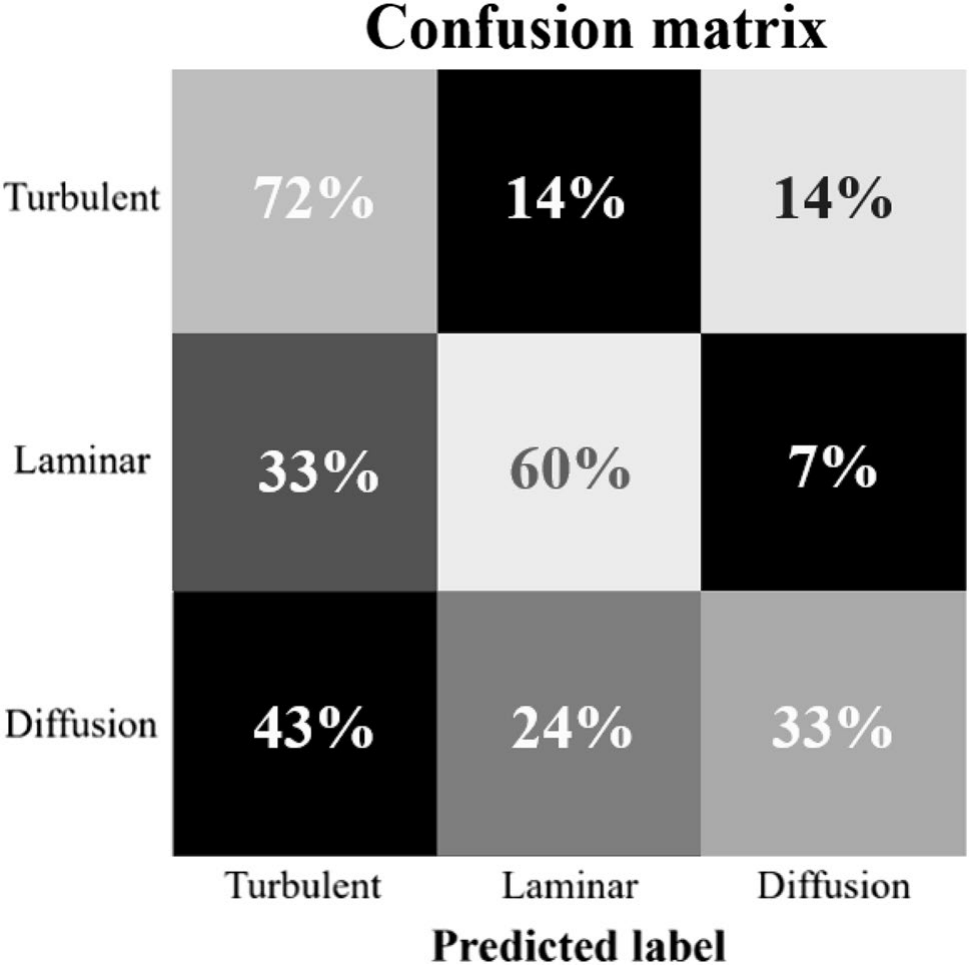
Confusion matrix. Results of the classification task using support vector machine on images characterized as feature vectors obtained from unsupervised deep learning applied on dried droplets of *Viscum album Quercus* 3× preparation produced through diffusion-based mixing, turbulent succussing, or laminar flow. These results provide more profound insights into the separability of the different mixing procedures.

Figure 5 depicts the results of the fully automated pattern classification employing the unsupervised pattern evaluation approach based on deep learning. The patterns obtained from *VaQ* 3×  could be correctly classified into the applied mixing procedures for the turbulently mixed variant for 72% of patterns, for the variant mixed using the laminar flow for 60% of patterns, and for the diffusion-based mixed control variant for 33% of patterns. The control variant was most frequently confounded with the turbulently mixed variant (43% of cases).

## Discussion

The findings of the present study suggest that the mixing method employed during the production of the *Viscum album Quercus L.* 3× (*VaQ* 3×) preparation affects the complexity of patterns formed in drying droplets (Supplement 1). A comparison of three differently mixed preparation variants reveals that turbulent flow (variant T) decreased the fractality of the patterns. Conversely, laminar flow (variant L) increased the fractality of the patterns compared to an unmixed, diffusion-based mixed sample (variant D).

In previous studies, the droplet evaporation method (DEM) was already proposed as a tool to test the influence of the number of vertical succussion strokes performed during the mixing of dilutions^4,5^; here, we demonstrate that not only the succussion number but also the mixing modality has an impact on the DEM patterns.

As indicated by other authors, the flow modality affects the behavior of molecules in suspension. Laminar flow leads to the alignment of longer molecules^14,15^, whereas turbulent flow promotes coagulation, particle formation, and oxidation of proteins, resulting in a more disordered and chaotic arrangement of suspended molecules^15^. Various studies have predicted^16^ and measured^17^ the influence of mixing on particle formation and crystallization processes. Additionally, it has been observed that flow regimes affect the shapes of particles formed in solutions, with simple-shaped particles formed under laminar flow and more complex particles under turbulent flow^18^.

At this point, we may hypothesize that in the experimentation presented here, the application of laminar mixing following each of the three decimal dilution steps of *VaQ* 3× likely contributed to a greater alignment of molecules in suspension and the creation of small and simple-shaped particles. Conversely, turbulent vertical succussions likely led to the agglomeration of molecules and increased formation of more complex particles and microbubbles^19^. In turn, during drying, droplets of the differently mixed *VaQ* 3× variants formed patterns of different complexity degrees. In variant L, the aligned molecules self-assembled into highly complex patterns, with the small and simple-shaped particles and low microbubble content not significantly disrupting the pattern formation process. Whereas, in variant T, the pattern formation process was likely hindered by the presence of agglomerated molecules, large and complex-shaped particles, and a high content of microbubbles, resulting in a decrease in pattern complexity. Further, it can be hypothesized that the changes induced by mixing were relatively stable and could not be altered or nullified by pipetting the differently mixed variants for droplet deposition on substrates.

Patterns resulting from the droplet evaporation method (DEM) and other methods based on evaporation-induced pattern formation are often evaluated solely through visual assessment, which can introduce subjectivity and bias into the results^20,21^. Utilizing deep learning for the evaluation of patterns formed in dried solution droplets has emerged as an effective alternative in various models^22–27^, offering rapid and objective image classification. In our study, we demonstrate that DEM images obtained from a *VaQ* 3× solution mixed using turbulent or laminar flow exhibit differences from a diffusion-based mixed control when subjected to semi- and fully-automated deep learning pattern classification^12,13^. Notably, all applied pattern evaluation approaches (i.e., visual inspection, computer-assisted analysis, and deep learning-based classification) yielded consistent conclusions regarding the impact of different mixing methodologies. Adopting deep learning facilitates the swift evaluation and comparison of large image databases for DEM patterns, offering substantial support for advancing further DEM applications^28–31^.

## Materials and methods

### Workflow

Three variants of *Viscum album Quercus* L. (*VaQ*) 3× (i.e., third decimal dilution prepared in decimal dilution steps, each followed by a succussion) were prepared using either (1) turbulent succussions by a machine performed for 2.5 min (variant T), (2) laminar flow induced by a series of handmade vortexes for 2.5 min (variant L), or (3) diffusion-based mixing (variant D). Variants T, L, and D were blinded and analyzed in a series of five droplet evaporation method (DEM) experiments, resulting in a total of 606 patterns. Each pattern was captured using a darkfield microscope at a magnification of 100×. The image database of 606 patterns was then analyzed using *ImageJ* software to assess grey-level distribution, texture, and fractality. Statistical analysis was conducted to compare the differences between the image evaluation parameters of the variants.

To ensure the stability of the experimental system, five systematic control experiments were conducted using only variant T, following the same experimental setup as the corresponding main experiments. The systematic control experiments yielded a total of 593 patterns. Insignificant results from these control experiments indicate a stable experimental system.

Additionally, the database from five main experiments was shared with the Pattern Analysis Lab at CINVESTAV, Mexico, and analyzed using supervised and unsupervised deep learning algorithms. The results of deep-leaning-based pattern evaluation are published elsewhere^12,13^; here, we provide a brief overview of the main outcomes.

### *Viscum album Quercus* L.

The utilization of plants in the present study adhered to international institutional guidelines. The *Viscum album Quercus* tincture was prepared by ISCADOR AG (Arlesheim, Switzerland). The plants were harvested from *Quercus robur* growing in natural habitats in Switzerland (owned by ISCADOR AG) and were identified by Mirio Grazi (Society for Cancer Research, Arlesheim, Switzerland). A voucher specimen (C.H. Quaresma 18.329) was deposited at the Herbarium of the Faculdade de Formação de Professores, Universidade Estadual do Rio de Janeiro, Brazil^32–34^.

### Potentization

Purified water according to Pharm. Eur. 9.4^7^ (“purified water in bulk”, X-SEPTRON LINE 10 VAL, BWT AQUA AG, Aesch, Switzerland) containing 2 × 10^−5^ g/ml NaCl and 2 × 10^−5^ g/ml KCl was used as the dilution medium. The salt addition served to enhance the pattern-forming capacities. In the first dilution step, a ratio of 1:20 was employed. Specifically, into each of the three 50ml capacity Erlenmeyer flasks, 1 ml of the *Viscum album Quercus* extract and 19 ml of the dilution medium were added. The contents of the first flask were gently stirred with a glass stirrer without inducing any vortex or foam formation. This flask was then closed with a tap and left undisturbed for 15 min (variant D, diffusion-based mixing). The remaining two flasks were also closed with taps; one was subjected to succussion on a succussion machine, performing vertical vigorous strokes for 2.5 min (variant T, turbulent mixing), while the other was mixed manually by repeatedly turning the flask in a circular movement to create a vortex and then allowing it to settle (variant L, laminar flow; see Supplement 1). The potentization to 2 × and 3× was carried out using dilution ratios of 1:10. For variants L and D, Erlenmeyer flasks with a capacity of 250 ml were filled up to 40 ml, whereas for variant T, 50 ml Erlenmeyer flasks were filled up to 30 ml. The choice of flask size and solution volume was determined experimentally to optimize vortex and turbulence formation for each variant.

### Droplet evaporation method

For each of the ten experiments (five main and five systematic control experiments), 12 microscope slides (76 × 26 mm, pre-cleaned, cut edges; Thermo Scientific, Gerhard Menzel B.V. & Co. KG, Braunschweig, Germany) were used. The slides were degreased by washing them with a dishwasher liquid and thoroughly rinsed under hot tap water following four consecutive purified water baths. Cleaned slides were wiped dry with a laboratory wiper (KIMTECH science, Kimberly-Clark Professional, Roswell, Canada) just before droplet deposition. In the main experiments, 3 μl droplets of variants T, L, and D were deposited on four slides each. Each slide contained two parallel rows, with each row having seven droplets. Droplets were deposited using a micro-pipette with a capacity of 2–20 μl (Eppendorf Research Plus, Eppendorf, Hamburg, Germany). For the systematic control experiments, all 12 slides were covered with droplets of variant T. Droplet desiccation occurred in an incubator (KBF 720, cooled incubator with controlled humidity system, WTB Binder Labortechnik GmbH, Tuttingen, Germany) with an inner plexiglass chamber covered with a semi-permeable cover and placed on a vibration-absorbing base. The 12 slides with droplets were positioned in the inner chamber, arranged in 4 rows of 3 slides each, following a quasi-randomization design to ensure uniform distribution of the tested T, L, and D variants within the chamber. The slides were left at 26 °C and 44% relative humidity until dry.

### Acquisition of patterns

The dried droplet residues were examined and photographed in darkfield at 100-fold magnification (100×) using an optical microscope (Zeiss Lab.A1; Carl Zeiss Microscopy GmbH, Jena, Germany) equipped with an attached camera (Moticam 5.0 MP; CMOS; Motic Electric Group Co., Ltd, Xiamen, China). The photographs were captured to encompass the dendritic structures that emerged within the droplet residues. Residues exhibiting disturbed pattern formation due to contaminating particles or edge effects on the slide were excluded from consideration. The images were saved in JPEG format with a resolution of 2592 × 1944 pixels. The five main experiments resulted in a total of 606 images (206, 196, and 204 for variants T, L, and D, respectively), while the five systematic control experiments produced a total of 593 images (198, 197, and 198 for the control groups of variant T, treated as control-T, control-L, and control-D, respectively).

### Computerized pattern evaluation

Image analysis was conducted using the software ImageJ (v. 1.50b)^35^ with installed plug-ins GLCM Texture and Frac-Lac^36^. The following steps were performed:

- Background extraction: all images underwent background extraction using a sliding paraboloid with a rolling ball radius set at 50 pixels. This process ensured a uniform background throughout the image database and reduced glares.
- Grey-level distribution analysis: Images were analyzed using the *ImageJ* tool "measure" to assess grey-level distribution.
- Texture analysis: After conversion into the 8-bit type, images were analyzed using the GLCM algorithm with distances between pixel pairs set to 4 pixels and angles of 90°. Parameters *ascending second moment, contrast, correlation, inverse difference moment,* and *entropy* were extracted to characterize texture.
- Fractal analysis: Images were resized to 500 × 375 pixels and converted into binary format. Fractal analysis was performed using Frac-Lac's DLC tool with the odd sizes scaling method and size limits for the grid caliber of 4–40 pixels. Parameters describing local connected fractal dimension, such as *box count fractal dimension, mass fractal dimension, fractal dimension of structures with the highest r2,* and *lacunarity*, were extracted.

Due to errors occurring in the image background after conversion to binary, some images were excluded from the fractal analysis. Specifically, for the main experiments, 4 images of variant T and 1 image of variant L were excluded. For the systematic control experiments, 14 images of variant control-D, 17 images of variant control-T, and 18 images of variant control-L were excluded.

### Statistical analysis

The data of the computerized image evaluation were transferred to Excel and analyzed by means of a two-way analysis of variance (CoStat, v. 6.311) (CoHort Software, Monterey, USA) at alpha = 0.05 with independent factors mixing method and day. The interaction between the factors was considered to assess the reproducibility within experiments performed on different days. Data distribution was checked visually; slight deviations from normality were irrelevant due to the central limit theorem. The global significance was determined using F-tests, whereas pairwise mean comparison was run two-tailed with the protected Fisher’s least significant difference test (pairwise comparisons were evaluated only if the global F-test was significant at p < 0.05). This procedure ensures a good safeguard against type I and II errors and thus balances well between false-positive and false-negative conclusions^37^.

### Deep-learning-based pattern evaluation

Supervised and unsupervised deep-learning-based methodologies for evaluating the DEM pattern database of the main experiments were developed for the purpose of this project; the protocols of these methodologies and obtained results are described in detail elsewhere^12,13^. In short: Images were subjected to background subtraction and converted to binary. From each image, patches were automatically selected in order to have the whole area covered with uniform structures and not contain any background, obtaining 705 patches from each mixing procedure of size 128 × 128 pixels. In the semi-supervised approach, based on their textures^38^, the patches were assigned to three pre-defined clusters (more fractal, medium fractal, and less fractal). We employed the DenseNet-121 architecture, utilizing a learning rate of 0.01 (describing the size of parameter updates), executing 30 epochs (i.e., iterations through the dataset), and employing a batch size of 32. In the unsupervised approach, which entails encoding texture information through generating a Deep Texture Representation matrix, the number of clusters was chosen automatically by the elbow method under a hierarchical clustering framework^39^ used to group the patterns obtained through the network. We leveraged the VGG-19 network (a specific neural network architecture whose weights have been previously learned) with a batch size of 16 (i.e., the number of samples processed simultaneously during training) and a learning rate of 0.01. We also conducted training over 30 epochs.

### Supplementary Information

Supplementary Video 1.

## Data Availability

The datasets generated and analyzed during the current study are available from the corresponding author upon reasonable request. Requests for programming codes should be addressed to M.C.
